# Bedside diagnostic laparoscopy to diagnose intraabdominal pathology in the intensive care unit

**DOI:** 10.1186/cc7730

**Published:** 2009-02-25

**Authors:** Adriano Peris, Stefania Matano, Giuseppe Manca, Giovanni Zagli, Manuela Bonizzoli, Giovanni Cianchi, Andrea Pasquini, Stefano Batacchi, Alessandro Di Filippo, Valentina Anichini, Paola Nicoletti, Silvia Benemei, Pierangelo Geppetti

**Affiliations:** 1 Intensive Care Unit of Emergency Department, Careggi Teaching Hospital and University of Florence, Viale Morgagni 85, 50139, Florence, Italy; 2 Department of General Surgery, Careggi Teaching Hospital and University of Florence, Viale Morgagni 85, 50139, Florence, Italy; 3 Clinical Pharmacology and Clinical Research Unit, Department of Pharmacology, University of Florence, Viale Pieraccini 6, 50139, Florence, Italy

## Abstract

**Introduction:**

Delayed diagnosis of intraabdominal pathology in the intensive care unit (ICU) increases rates of morbidity and mortality. Intraabdominal pathologies are usually identified through presenting symptoms, clinical signs, and laboratory and radiological results; however, these could also delay diagnosis because of inconclusive laboratory tests or imaging results, or the inability to safely transfer a patient to the radiology room. In the current study we evaluated the safety and accuracy of bedside diagnostic laparoscopy to confirm the presence of intraabdominal pathology in an ICU setting.

**Methods:**

This retrospective study, carried out between January 2006 and June 2008, evaluated the diagnostic accuracy of bedside diagnostic laparoscopy performed on patients with a suspicion of ongoing intraabdominal pathology. Clinical indications for bedside diagnostic laparoscopy were: ultrasonography (US) images of gallbladder distension or wall thickening of more than 3 to 4 mm, with or without pericholecystic fluid; elevation of laboratory tests (bilirubin, transaminases, myoglobin, lactate dehydrogenase, creatine phosphokinase, gamma-glutamyltransferase); high level of lactate/metabolic acidosis; CT images inconclusive for intraabdominal pathology; or inability to perform a CT scan. Patients did not undergo bedside diagnostic laparoscopy if they presented clear indications for open surgery, coagulopathy, abdominal wall infection or high intraabdominal pressure.

**Results:**

Thirty-two patients underwent bedside diagnostic laparoscopy (Visiport Plus, Autosuture, US), 14 of whom had been admitted to the ICU for major trauma, 12 for sepsis of unknown origin and 6 for complications after cardiac surgery. The procedure was performed on an average of eight days after ICU admission (95% confidence interval = 5 to 15 days) and mean procedure duration was 40 minutes. None of the procedures resulted in complications. Bedside diagnostic laparoscopy was diagnostic for intraabdominal pathology in 15 patients, who subsequently underwent surgery, except in two cases of diffuse gut hypoperfusion. Diagnosis of cholecystitis was obtained in seven cases: two were treated with laparotomic cholecystectomy and five with percutaneous gallbladder drainage positioning.

**Conclusions:**

Bedside diagnostic laparoscopy represents a safe and accurate technique for diagnosing intraabdominal pathology in an ICU setting and should be taken into consideration when patient transfer to radiology or the operating room is considered unsafe, or when routine radiological examinations are not conclusive enough to reach a definite diagnosis.

## Introduction

Acute life-threatening intraabdominal pathologies, such as intestinal perforation, ischaemia, sepsis, post-traumatic haemorrhage, pancreatitis and biliary diseases, represent a diagnostic challenge for clinicians. Additionally, intraabdominal pathologies may occur as complications of long-term intensive care unit (ICU) hospitalisation. In fact, prolonged fasting or parenteral nutrition, mechanical ventilation and high-dose opioid analgesics are definite risk factors for acalculous cholecystitis which, in critically ill patients, is often complicated by gangrene or perforation, leading to a long recovery [1-3].

Overall, abdominal complications in patients in the ICU are reported to be strongly associated with an increased risk of death: the mortality rate for abdominal sepsis is about 30 to 50% [4], and rises to 70% in patients post-cardiac surgery [5]. Prompt diagnosis, followed by causal therapy, is the only way to increase a patient's chance of survival.

Abdominal symptoms are often hidden by the presence of deep sedation and/or analgesia, so laboratory tests (e.g. leucocytes count, procalcitonin, lactate or specific enzymes plasma levels), arterial blood gas analysis and, above all, radiological findings, become the key to a correct diagnosis of intraabdominal pathology. However, radiological examinations are not always possible or accurate enough to make a unambiguous diagnosis. For instance, computerised tomography (CT) scan has high diagnostic sensitivity for a series of intraabdominal pathologies, but requires patient transfer to the radiology room [4]. As an alterative, ultrasonography (US) can be performed at the bedside and has greater accuracy for biliary tract pathologies, even though it is an operator-dependent procedure [4,6].

Bedside diagnostic laparoscopy has been proposed as a valuable diagnostic option in the ICU for patients with sepsis of unknown origin or multi-organ failure with high suspicion of intraabdominal pathology [7-9]. Bedside diagnostic laparoscopy is minimally invasive and less expensive than exploratory laparotomy. In this regard, a recent study emphasised the potential advantage of bedside diagnostic laparoscopy in critically ill patients, with evidence levels 2 and 3, especially when acalculous cholecystitis or ischaemic bowel disease are suspected [10]. This study, however, highlighted the need for more extensive, appropriate examination. Thus, the aim of the current investigation was to evaluate the safety and diagnostic accuracy of bedside laparoscopy in the identification of intraabdominal pathology in critically ill patients.

## Materials and methods

### Data collection

We retrospectively studied patients admitted to the ICU of the Careggi Teaching Hospital, Florence, Italy, who had undergone bedside diagnostic laparoscopy between January 2006 and June 2008. Patients' demographic and clinical characteristics, admission diagnosis, laparoscopic diagnosis and treatments administered after bedside diagnostic laparoscopy were collected in an ICU database (FileMaker Pro 5.5v2; FileMaker Inc, Santa Clara, CA, USA). The severity of illness was estimated using the Simplified Acute Physiology Score II (SAPS II) at the time of ICU admission.

Bedside diagnostic laparoscopy was performed during hospitalisation if clinical signs and/or laboratory/imaging findings were suggestive, but not conclusive, for intraabdominal pathology. Indicators considered in the execution of bedside laparoscopy were: US images of gallbladder distension or wall thickening more than 3 to 4 mm, with or without pericholecystic fluid; persistent elevation of laboratory tests (bilirubin, transaminases, myoglobin, lactate dehydrogenase, creatine phosphokinase, gamma-glutamyltransferase); high level of lactate/metabolic acidosis; or CT scan images not conclusive for intraabdominal pathology. In addition, the inability to perform a CT scan because of the patient's critical condition was also considered a valid reason to execute the procedure.

Patients did not undergo bedside diagnostic laparoscopy if they possessed at least one of the following characteristics: clear indication for open surgery; previous diagnosis of coagulopathy; evidence of abdominal wall infection; or high intraabdominal pressure (above 15 mmHg), evaluated by measuring urinary bladder pressure (AbViser, Wolfe Tory Medical Inc., Salt Lake City, Utah, USA). The study was performed in accordance with the principles of the Declaration of Helsinki and was approved by the Local Ethics Committee, which waived the need for written informed consent because of the retrospective nature of the study.

### Operative technique

Bedside laparoscopy was performed with Visiport Plus Optical Trocar (5 to 11 mm) and Versaport Plus Cannula (Covidien Autosuture, Mansfield, MA, US), placed on a mobile tower. All procedures were performed in a isolated single bedroom of the ICU ward by GM (who performed all the procedures included in this study), a nurse from the operating room, one of the anaesthetists on duty (with a colleague available when needed) and two ICU nurses. All the staff present in the room wore protective clothing, a surgical cap, gloves and a surgical mask. Sterility was warranted by adherence to routine operating-room protocols and sterilisation of the operating site with povidone-iodine (10%).

The anaesthesiologist on duty directed the administration of total intravenous anaesthesia, ventilation and haemodynamic support. General anaesthesia was induced by a bolus of propofol (1 to 2.5 mg/kg), midazolam (0.15 to 0.2 mg/kg) or ketamine (0.5 to 1 mg/kg) and remifentanil (0.5 to 1 μg/kg/minute) or fentanyl (1 to 2 μg/kg), followed by infusion of propofol (4 to12 mg/kg/hour) and fentanyl (25 to 100 μg) or remifentanil (0.5 to 1 μg/kg/minute); neuromuscular block was achieved with atracurium (0.5 to 0.7 mg/kg). With the patient in a supine, Trendelenburg or anti-Trendelenburg position to obtain the most appropriate laparoscopic view (e.g. diaphragmatic exploration), trocar was placed into the paraumbilical region. In two patients who underwent prior laparotomic surgery, trocar was inserted through a portion of the laparotomy incision, as previously described [8]. Pneumoperitoneum was achieved by inflating the abdominal cavity with carbon dioxide at 8 to 15 mmHg.

During the procedure, patients were mechanically ventilated (volume-controlled, 6 to 10 ml/kg; inspiratory oxygen fraction (FiO_2_) 40 to 70%; Positive End-Expiratory Pressure (PEEP) 6 to 10 cmH_2_O) and invasive arterial blood pressure, electrocardiogram, pulse oximetry and end-tidal carbon dioxide were constantly monitored. When required, haemodynamic support was established by noradrenaline (0.1 to 1 μg/kg/minute) and/or dobutamine (2 to 6 μg/kg/minute) infusion.

## Results

### Overall population

During the 30-month study period, 32 patients fulfilled the indication criteria and underwent bedside diagnostic laparoscopy: 14 patients were admitted for major trauma, 12 for sepsis of unknown origin and six for complications due to prolonged extracorporeal circulation during cardiac surgery (Table 1). On average, bedside diagnostic laparoscopy was performed within eight days (range 5 to 15 days) of ICU admission and lasted 40 minutes (average data). Metabolic and haemodynamic parameters were not affected by the procedure, including anaesthesia (data not shown). No complication was reported. In 46.9% of the study participants (n = 15), bedside diagnostic laparoscopy confirmed the suspicion of intraabdominal pathology. None of the enrolled patients reported post-procedure abdominal wall infections.

**Table 1:** Demographics, admission diagnosis, severity of illness and mortality rate of the study population

**Total number of patients**	32
**Age (years) (mean ± SD)**	58.3 ± 20.1

**Male sex, % (N)**	71.9% (23)

**Admission diagnosis**	
**trauma, % (N)**	43.8% (14)
**sepsis, % (N)**	37.5% (12)
**post-cardiac surgery, % (N)**	18.7% (6)

**SAPS II score (mean ± SD)**	46.71 ± 9.1

**Mortality, % (N)**	34.4% (11)

Data are expressed as percentage of the overall population.

SAPS II = Simplified Acute Physiology Score II; SD = standard deviation.

### Trauma patients

Fourteen polytraumatized patients underwent bedside diagnostic laparoscopy: 11 were negative and three were found to be positive for acalculous cholecystitis and treated with percutaneous gallbladder drainage; in one patient with negative bedside diagnostic laparoscopy exploration, a radiological suspicion of right diaphragmatic injury was excluded (Table 2).

**Table 2:** Diagnostic indications of bedside diagnostic laparoscopy, treatment delivered and final outcome

			**Outcome**	
			
**Diagnostic group (N)**	**Results of BDL (N)**	**Therapeutic approach after procedure**	**survived**	**deceased**
**Trauma (14)**	Negative (11)	Conservative	8	3
	
	Acalculous cholecystitis (3)	Percutaneous gallbladder drainage	2	1

**Sepsis (12)**	Negative (6)	Conservative	3	3
	
	Purulent peritonitis with colic perforation (2)	Colostomy, anastomosis and VAC therapy	1	1
	
	Purulent peritonitis with gut ischaemia (1)	Ileostomy, anastomosis and VAC therapy	1	
	
	Purulent peritonitis without other evidences (3)	VAC therapy	2	1

**Post-cardiac surgery (6)**	Gangrenous cholecystitis (4)	Laparotomic cholecystectomy (2)	2	0
		
		Percutaneous gallbladder drainage (2)	2	0
	
	Diffuse gut hypoperfusion (2)	Conservative	0	2

Survived patients were defined as patients discharged alive from the Hospital.

BDL = bedside diagnostic laparoscopy; VAC = vacuum-assisted closure.

### Septic patients

Among the 12 patients admitted for sepsis of unknown origin, bedside diagnostic laparoscopy was able to detect an ongoing purulent peritonitis in six patients that were negative on the peritoneal fluid microbiological cultures. Subsequent open laparotomy in the operating room detected two colic perforations and one segmental ischaemia of the distal ileum. In three patients, diagnosis of purulent peritonitis was confirmed without other evidence of pathology (Table 2). In all cases, the abdominal wall was left open after the procedure and a vacuum-assisted closure devise (Kinetic Concepts Inc., San Antonio, TX, USA) was positioned for 48 to 72 hours, to prevent the development of abdominal compartment syndrome. For the six patients with negative exploration, bedside diagnostic laparoscopy was able to exclude an abdominal source of sepsis.

### Post-cardiac surgery patients

Among the six patients admitted after cardiac surgery, four had a positive result for gangrenous cholecystitis. Two subjects were treated with laparoscopic cholecystectomy in the operating room, and two with percutaneous gallbladder drainage. Two post-surgical patients had diffuse gut hypoperfusion and died of multi-organ failure. All four surviving patients treated for cholecystitis were discharged from the hospital (Table 2).

## Discussion

In critically ill patients, the evaluation of intraabdominal pathology based on clinical symptoms and signs might be unreliable, because abdominal pain and tenderness are frequently concealed by sedation or deep anaesthesia. For this reason, radiological analyses are essential to detect intraabdominal pathology but they can be ambiguous or not possible. When the patient is too unstable to be moved safely, US is the standard bedside examination but it has disadvantages, such as the operator-dependent results and extensive patient preparation [3]. Moreover, the results are not always conclusive [1,11,12].

Bedside diagnostic laparoscopy may facilitate the diagnosis of intraabdominal diseases. To our knowledge, following the 1989 survey by Iberti and colleagues [13], 13 studies have investigated the diagnostic indications of bedside diagnostic laparoscopy in different critically ill patients, including septic, traumatised and post-surgical patients [5,8,9,12,14-22] (Table 3). These studies reported the high diagnostic accuracy of bedside diagnostic laparoscopy for intraabdominal diseases, but not for pancreatitis, retroperitoneal or inner-cavity pathologies [10]. Nevertheless, one case report showed how this procedure, along with biopsy, was useful to obtain a rapid diagnosis of retroperitoneal malignancy [21]. Recognised advantages of bedside diagnostic laparoscopy are the possibility of avoiding unnecessary open laparotomic exploration and to reduce the risks of intrahospital transfers. Complications related to the transportation of critically ill patients include haemodynamic instability, respiratory distress, airway obstruction, artificial airway or intravenous line removal. All these events can severely increase the morbidity and mortality of critically ill patients [23].

**Table 3:** Summary of bedside diagnostic laparoscopy in the intensive care unit reviewed.

**Author (year)**	**ICU population studied**		**Results of bedside laparoscopy**		**Complications (N)**
	
	**Pathology (N)**	**Total N**	**Positive**	**Findings (N)**	
Bender and Talamini (1992) [14]	Severe burn (1) Thoracic surgery (1)	2	1	Acalculous cholecystitis	

Forde and Treat (1992) [15]	Cardiac arrest (3) Various medical diseases (7)	10 (9 bedside, not specified which of them)	4	Peritonitis (4)	Intraperitoneal haemorrhage (1)

Brandt and colleagues (1993) [12]	Trauma/burns (9) Cardiac/vascular surgery (6) Acute malignancy (4) Cardiac/respiratory arrest (3) Renal failure/sepsis (1)	25	12	Intestinal ischaemia (6) Gangrenous cholecystitis (4) Perforated caecum (l) Ruptured spleen (1)	

Brandt and colleagues (1994) [16]	Trauma	9 (1 bedside)	1	Gangrenous cholecystitis	

Almeida and colleagues (1995) [17]	Blunt trauma (8) Leg gunshot wound (1) Cardiac surgery (1)	10 (6 bedside)	3	Gangrenous cholecystitis (4) Distended gallbladder (1)	

Orlando and colleagues (1997) [18]	Cardiac surgery (19) Vascular surgery (2) General surgery (5)	26	16	Acute cholecystitis (10) Mesenteric ischaemia (5) Perforation (1)	

Walsh and colleagues (1998) [19]	Cardiac failure (4) Sepsis (3) Pneumonia (2) Cardiac surgery (1) Pulmonary failure (2)	12	5	Intestinal ischaemia (2) Thickened terminal ileum (1) Sigmoid diverticulitis (1) Peritonitis (1)	Transient bradycardia during procedure (1)

Kelly and colleagues (2000) [20]	Sepsis of unknown origin (14)	14	5	Intestinal ischaemia (3) Cholecystitis (2)	

Rosin and colleagues (2001) [21]	Sepsis after cardiac surgery (1) Sepsis after neurosurgery (1) Cardio-respiratory failure (1) Malignancy (1)	4	2	Viscus perforation (1) Abdominal abscess (1)	

Pecoraro and colleagues (2001) [8]	General surgery (4) Sepsis (3) Malignancy (2) Other (2)	11	8	Fibrinous or purulent exudates (3) Tumour (2) Intestinal ischaemia (1) Fistula (1) Cirrhosis (1)	

Gagne and colleagues (2002) [9]	Medical Surgical Trauma (numbers not specified)	19	6	Extensive mesenteric ischemia (3) Intestinal ischaemia (1) Gangrenous cholecystitis (1) Suggestive bowel ischaemia (1)	Gallbladder perforation (1) Ascitic leak from trocar site (1)

Hackert and colleagues (2003) [5]	Major cardiac surgery with extracorporeal circulation (17)	17	10	Colonic ischaemia (6) Acute cholecystitis (3) Fibrinous peritonitis (1)	Colonic perforation (1)

Jaramillo and colleagues (2006) [22]	Sepsis of unknown origin (13)	13	9	Intestinal ischaemia or necrosis (6) Acalculous cholecystitis (2) Colonic perforation (1)	

The use of bedside diagnostic laparoscopy has also been proposed in post-traumatic intraabdominal injuries, to facilitate a faster diagnosis in the emergency room. Its use in this setting has been extensively analysed by Stefanidis and colleagues in a recent review [10]. Bedside diagnostic laparoscopy is a minimally invasive procedure with a low reported complication rate, ranging from 1 to 9% of patients [5]. The most severe procedures-related complications were visceral perforation, pneumoperitoneum-induced bradycardia, intraperitoneal haemorrhage and post-procedure ascitic leak from trocar site [9,5,19] (Table 3). In our series of 32 cases, bedside diagnostic laparoscopy prevented open laparotomy in 17 subjects, 64% (n = 11) of whom were subsequently discharged in good clinical condition (Table 2). No complication of any origin or nature was observed. This high level of safety and accuracy could result from a strict adherence to our procedure protocol. In our experience, the positive outcome of bedside diagnostic laparoscopy can be associated with three major factors: cooperation among anaesthesiologists and the surgeon in the decision-making of whether to perform a bedside laparoscopy; single-bed isolated room setting, that guarantee an optimal operating-room-like environment; and daily emergency surgery technical skills of surgeon. As the level of intra-peritoneum pressure is the most critical intra-procedure parameter, we also confirm [10] and suggest a set up in the range of 8 to 15 mmHg, because this is usually well-tolerated and does not compromise mechanical ventilation or the haemodynamic parameters in critically ill patients.

When considering the effectiveness of this procedure by the main categories of diagnosis, in patients with sepsis of unknown origin, bedside diagnostic laparoscopy may be regarded as a good diagnostic tool [10]. Percentages of patients who avoided open laparotomy range from 30 [22] to 65% [20] (Table 3), and we showed that 50% of our septic patients obtained a bedside laparoscopy diagnosis followed by causal therapeutic intervention (Table 2). It should be emphasised that none of our patients who had a laparoscopic diagnosis of purulent peritonitis, tested positive in the peritoneal fluid microbiological cultures. Although this study was not designed to evaluate the value of diagnostic peritoneal lavage, our data do not encourage the use of this technique to exclude abdominal septic foci.

Bedside diagnostic laparoscopy should be taken into consideration especially in patients who have undergone open-heart surgery, in whom intraabdominal pathology complications are uncommon but potentially fatal [5,24]. Although performed on a small sample, we found high accuracy in diagnosing intraabdominal pathologies in patients post-cardiac surgery, leading to the correct identification and treatment of cholecystic pathologies (Table 2).

The incidence of acalculous cholecystitis in critically ill patients is high, because it is strongly associated with systemic inflammatory response syndrome, sepsis, abdominal/cardiac surgery, prolonged fasting and opioid administration [2,25]. The reported accuracy of bedside laparoscopy in the diagnosis of cholecystitis, gut perforation and intestinal ischaemia appears excellent (Table 3), even when radiological assessments (US, CT scan) produced false-negative results. In this regard, Brandt and colleagues [16] reported that in nine trauma patients, US and CT scan had an accuracy rate of 57% and 66%, respectively, whereas laparoscopies, although performed at the bedside of just one patient, did not produce a false-positive or false-negative diagnosis. One false-negative result was reported by Orlando and Crowell in a case series of 26 bedside laparoscopy procedures, with an initial diagnosis of viscus perforation and subsequent CT-scan evidence of pancreatitis [18]. In accordance with Gagne and colleagues [9] and, more recently, Jaramillo and colleagues [22], we found that bedside diagnostic laparoscopy was extremely effective for the diagnosis of acalculous cholecystitis in ICU patients, enabling the avoidance of open surgical exploration and, in some cases, permitting a conservative treatment (Table 2). Although a recent review underlined the diagnostic value of diagnostic peritoneal lavage for acalculous cholecystitis [4], Walsh and colleagues reported a low accuracy of diagnostic peritoneal lavage in revealing gallbladder pathologies, except in cases of acute perforation and consequent peritonitis [19].

## Conclusions

Our results indicate the advantages of the use of bedside diagnostic laparoscopy in the ICU setting. Bedside diagnostic laparoscopy should be contemplated anytime there is the suspicion of intraabdominal pathology based on suggestive, but not conclusive, laboratory and radiological results, or in the case of the inability to transfer a critically ill patient to the radiology department.

## Key messages

• Bedside diagnostic laparoscopy represents an effective diagnostic option to uncover intraabdominal pathology, especially if acalculous cholecystitis is suspected.

• It might be considered in unstable patients for whom transportation to radiology or the operating room could be unsafe.

• Patients who underwent open-heart surgery should be electively considered for bedside diagnostic laparoscopy if clinicians have high suspicion of intraabdominal pathology.

• In traumatised patients, bedside diagnostic laparoscopy seems to be effective in diagnosis/exclusion of acalculous cholecystitis.

## Abbreviations

CT: computerized tomography; FiO_2_: inspiratory oxygen fraction; ICU: intensive care unit; PEEP: positive end-expiratory pressure; SAPS: Simplified Acute Physiology Score; US: ultrasound.

## Competing interests

The authors declare that they have no competing interests.

## Authors' contributions

AdP, MB and AnP designed the study. AdP, MB, GC, AnP, ADF and GZ reviewed the literature. SM, VA, GC, StB and SiB collected and elaborated data. GM performed all surgical interventions. SM, GZ, AdP, PN, StB, SiB and PG wrote and revised the manuscript. All authors have seen and approved the final revised version.
